# Subdeltoid lipoma causing shoulder impingement syndrome – a case report

**DOI:** 10.1590/S1679-45082014RC2934

**Published:** 2014

**Authors:** Mario Lenza, Miguel Vicente Lenza, Eduardo da Frota Carrerra, Mario Ferretti

**Affiliations:** 1 Hospital Israelita Albert Einstein, São Paulo, SP, Brazil.; 2 Hospital Nove de Julho, São Paulo, SP, Brazil.

**Keywords:** Shoulder impingement syndrome, Shoulder joint, Lipoma, Rotator cuff, Pathology, surgical, Case reports

## Abstract

The impingement syndrome is defined by the compression of the rotator cuff tendons against the coracoacromial arch. Several factors contribute to this condition and they are classified as structural or functional factors. The former are changes in the coracoacromial arch, proximal humerus, bursa and rotator cuff, and the latter are related to the mechanism of the upper limb by means of synchronized activity and balanced between the rotator cuff and scapular girdle muscles. The authors report here a case of parosteal lipoma of the proximal humerus, located between the muscles deltoid, teres minor and infraspinatus causing clinical signs of impingement. It is a rare occurrence, characterized as a structural cause for the onset of this symptom.

## INTRODUCTION

Lipomas are the most common benign tumors of the connective tissue, made up of encapsulated nodules of fat that may contain fibrous tissue. Microscopically they are composed of mature adipose cells, without any evidence of cellular atypia.^(1, 2)^ These tumors are more common in females between 30 and 60 years of age, clinically they present as a soft circumscribed, movable generally painless mass.^(2)^

According to Enzinger and Weiss,^(3)^ there are two types of lipomas: superficial and deep. The superficial lipomas are more commonly found in the shoulder, neck and abdominal region. The deep lipomas occur less frequently, and are located in the anterior mediastinum, chest wall and retroperitoneal region, and they are divided into the following types: intramuscular, intermuscular, parosteal and intra-osseous.

Among the deeply located lipomas, the intramuscular and intermuscular types are the most common; parosteal lipoma is infrequent; and the intraosseous is rare.^(1)-4)^ The parosteal lipoma is a deep lipoma that grows in an exophytic manner in the periosteum, and can produce bone erosion, neural compression or focal cortical hyperostosis. It is a rare, slow growing histologically benign tumor, comprising 0.3% of all lipomas.^(1, 3, 4)^

When studying 18,677 mesenchymal tumors, Kransdorf^(5)^ observed that 16% were lipomas – in that, 1.3% was intramuscular and 2.6% were located in the shoulder and axilla, in a deep site.

The present case describes a patient with diagnosis of parosteal lipoma of the proximal humerus inducing the clinical signs of impingement syndrome. Searching the literature, no similar case was found.

## CASE REPORT

An 82-year-old female patient, with history of pain in the left shoulder for two years, reported more intense pain over the last two months. Upon onset of symptoms she sought medical treatment. The diagnosis of supraspinatus tendonitis was made based on the clinical examination and ultrasonography. The patient was prescribed conservative treatment as physical therapy (analgesia and kinesiotherapy). For the following 18 months, the symptoms alternated between periods of relief and intensification.

Over the last 2 months, her pain markedly increased, with loss of functional capacity of the left upper limb affecting her daily activities. Elevation of this limb caused intense pain and at night, pain woke her up many times.

On physical examination, the patient presented mild hypotrophy of the deltoid and on dynamic inspection, active elevation was 90° and passive 160°. Reduction in supraspinatus muscle strength during the empty can test,^(6)^ and a positive Neer sign^(7)^ were verified. Other tests in physical examination and the range of motion in lateral and medial rotation compared to the opposite side were assessed and considered normal.

After an injection of 10mL xylocaine in the subacromial space, pain disappeared and the patient was able to move her shoulder normally (positive Neer test).^(7)^

Anteroposterior radiographs of the left shoulder, axillary and supraspinatus tunnel (outlet view) were ordered. A type-II acromion, according to Bigliani^(8)^ classification, was observed.

**Figure 1:**
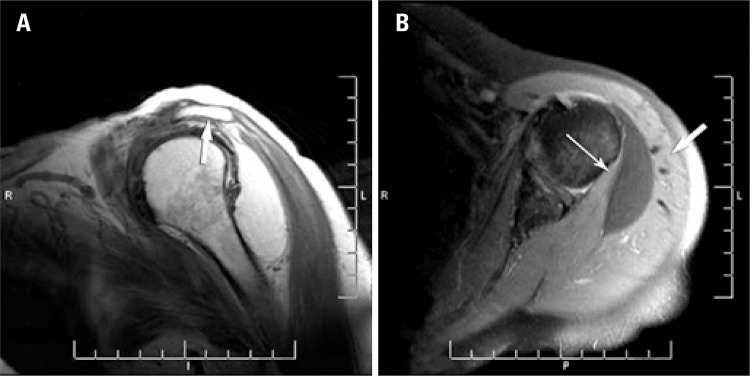
. (A) Sagittal view showing subdeltoid lipoma not invading the subacromial space and tapering of the supraspinatus tendon (arrow). (B) Axial view showing the lipoma between the teres minor (narrow arrow) and deltoid (wide arrow) muscles

The magnetic resonance image (MRI) of the left shoulder (Figures 1A and 1B) revealed supraspinatus tendonitis, with no rupture of the rotator cuff tendon and a massive soft tissue tumor (probably a lipoma) measuring 6.2x5.8x2.4cm, visible between the bellies of the deltoid, teres minor and infraspinatus muscles.

**Figure 2:**
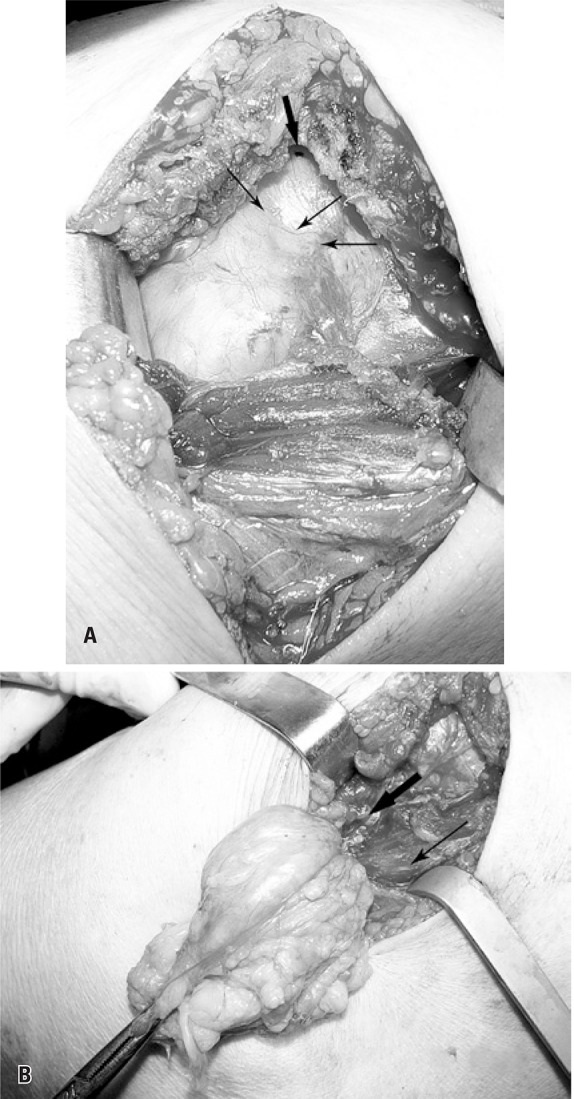
. (A) Intraoperative image demonstrating that the lipoma (narrow arrow) does not invade the subacromial space (wide arrow). (B) Lipoma adhering to the humerus, anteriorly to teres minor tendon (narrow arrow) and infraspinatus (wide arrow) muscles

Removal of the tumor was indicated, and it was performed by posterior approach. The soft tissue tumor did not invade the subacromial space (Figure 2A). Its upper extremity was situated at the height of the posterior acromion and the lower extremity was attached to the periosteum of the proximal humerus, anteriorly to the tendon insertions of the infraspinatus and teres minor muscles (Figure 2B). The vessels present at this site and penetrating the tumor capsule were cauterized.

**Figure 3:**
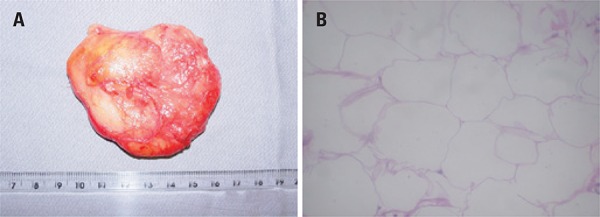
. (A) Image of the surgical specimen. (B) Photomicrograph of the histological section of the lipoma (hematoxylin and eosin, 200x)

The tumor was resected by a marginal excision (as “shelling out the tumor”), and the deltoid reinserted in the posterior acromion. The resected mass measured 7.0x5.5x1.5cm (Figure 3A). The histological examination report was parosteal lipoma (Figure 3B).

In the postoperative period the patient remained with thoracobrachial immobilization for the first 6 weeks, only making passive movements of lateral and medial rotation. This was followed by mild active exercises without resistance, and after 2 months, she began on muscle strengthening. The patient has been asymptomatic 26 months after surgery.

## DISCUSSION

The normal function of the shoulder muscle structure depends on specific requirements such as: preserved integrity and tonus of the rotator cuff muscles (spraspinatus, infraspinatus, teres minor and subscapular), normal capsular laxity, integral coracoacromial arch, thin and lubricating bursae, and concentricity of the glenohumeral and coracoacromial rotational spheres. Alterations of this intricate mechanism are the most common causes of shoulder pain problems.^(9)^

The shoulder is shaped by four anatomic barriers (space circumscribed by a thick wall of collagen of the fascias and tendons, and the bones): the scapula with its muscles; the clavicle; the proximal humerus; and the deltoid muscle. In these spaces, the tumors grow centrifugally when in contact to these anatomic barriers.^(2)^ In the case reported, the lipoma was of the parosteal type, growing in the compartment between the deltoid, infraspinatus, teres minor muscles and the humerus.

The tumor of this patient increased the pressure in this compartment upon muscle contraction, altering the concentric relation of the rotational spheres. In its movement arch, the joint between the humerus and scapula is formed by two concentric spheres.

The humeral sphere is represented by the head of the humerus (with a smaller radius) and the sphere of the coracoacromial arch formed by the lower surface of the anterior acromium and the coracoacromial ligament (with a larger radius). Both spheres have the same rotational center and together maintain the best condition of stability for the shoulder and for the available surface for transfer of the scapular load.^(9)^

The range of motion in arm elevation in the scapular plane is zero to 180°. In the initial stage of movement up to 90°, fixation of the scapula occurs over the chest wall by contraction of the trapezius and anterior serratus muscles. The muscles of the rotator cuff lower and fix the head of the humerus in the glenoid cavity, and, at this time, the deltoid muscle, with predominance of its mid- and posterior portions, acts by raising the arm. When the upper limb reaches an elevation of 90°, the greater tuberosity in order to move under the coracoacromial arch, requires the external rotation of the humerus, produced by the contration of the infraspinatus and the teres minor muscles. These muscles act in synchrony producing a smooth and coordinated motion.^(9)^ In this case, when raising her arm, the synchrony in contraction of the deltoid, infraspinatus and teres minor muscles is interrupted, due to the lipoma interposed between them. The motion loses its coordination causing the impact of the greater tuberosity against the coracoacromial arch.

Resection of the tumor restored the concentricity of the two spheres of movement (head of the humerus and anterior acromion and coracoacromial arch) during the lateral rotation in the final degrees of elevation, due to the recovery of congruity of the glenohumeral joint in its entire movement arch, leading to relief of symptoms of impingement syndrome. A limitation of this case report was the lack of electroneuromyography requested to patient; this diagnostic exam could detect a compression of axillary nerve in the quadrangular space that mimics the symptoms of patients.

To find articles evaluating patients with subacromial syndrome related to lipoma, we searched MEDLINE via PubMed (1966 to July 2013), EMBASE (1980 to 2013 Week 20), and LILACS (1982 to July 2013). There was no language or publication status limitation to our search. We also checked references of articles and reviews for other potentially relevant studies. Our search strategy combined the specific terms (“shoulder impingement syndrome” [Mesh] OR “shoulder joint” [Mesh] OR “rotator cuff” [Mesh]) AND “lipoma” [Mesh].

The search strategy (completed in July 2013) identified a total of 36 records from the following databases: PubMed (19), EMBASE (11), LILACS (6). The search resulted in the identification of six articles that evaluated patients with shoulder condition associated with lipoma.^(10)-15)^

Rohrbough and Jobe^(10)^ reported a case of lipoma located in the subdeltoid region, that produced symptoms of glenohumeral instability. Nisolle et al.^(11)^ presented a 44-year-old man with bursal *lipoma arborescens* of the right shoulder associated with a rotator cuff tear. Relwani et al.^(12)^ described a case of a 52-year-old woman with classic signs of shoulder impingement and the patient had an anterior subacromial lipoma causing the symptoms. Two case-report articles illustrated two cases of a 45-year-old man and a 51-year-old man with signs and symptoms consistent with impingement syndrome whose etiology was intramuscular lipoma involving the supraspinatus muscle.^(13, 14)^ In addition, a recent case report presented a 38-year-old male patient with symptoms mimicking both glenohumeral instability and subacromial impingement^(15)^ due a subdeltoid intermuscular lipoma. In all cases the symptoms disappeared after surgery and physical therapy.

We considered our search strategy comprehensive, with no language restrictions applied. It also included handsearching (grey literature) and searches for ongoing and recent narrative reviews and case reports. However, it is possible that we have neglected some potential studies describing a similar case. Therefore, to our knowledge, the present case illustrated in this article is an atypical case of a parosteal lipoma of the proximal humerus that caused signs and symptoms of subacromial impingement. The mechanism that trigger the symptoms was preoperatively diagnosed by the MRI test.
