# Supplementary material for: Subdeltoid lipoma causing shoulder impingement syndrome – a case report
Source: Einstein (Sao Paulo). 2014 Jul-Sep;12(3):351–4. doi: 10.1590/S1679-45082014RC2934 (PMC4872949; doi:10.1590/S1679-45082014RC2934)
Supplement: Translation

# Translation: Lipoma subdeltoídeo como causa de síndrome do impacto no ombro – um relato de caso

**Authors:** Mario Lenza, Miguel Vicente Lenza, Eduardo da Frota Carrerra, Mario Ferretti

**Affiliations:** 1 Hospital Israelita Albert Einstein, São Paulo, SP, Brasil.; 2 Hospital Nove de Julho, São Paulo, SP, Brasil.

**Keywords:** Síndrome de colisão do ombro, Articulação do ombro, Lipoma, Bainha rotadora, Patologia cirúrgica, Relatos de casos

## Abstract

A síndrome do impacto é definida pela colisão dos tendões dos músculos que formam o manguito rotador contra o arco coracoacromial. Vários fatores concorrem para essa doença e são classificados como estruturais ou funcionais. Os primeiros são alterações do arco coracoacromial, úmero, bursa e manguito rotador; os fatores funcionais estão relacionados com o mecanismo de elevação do membro superior, por meio da atividade sincronizada e equilibrada entre o manguito rotador e os músculos da cintura escapular. Os autores relatam aqui, o caso de um lipoma parosteal do úmero proximal, situado entre os ventres musculares do deltoide, redondo menor e infraespinhal ocasionando os sinais clínicos da síndrome do impacto. Trata-se de uma ocorrência rara, caracterizada como uma causa estrutural para o desencadeamento dessa sintomatologia.

## INTRODUÇÃO

Lipomas são os tumores de tecido conjuntivo mais frequentes, formados por nódulos encapsulados de gordura que podem conter tecido fibroso. À microscopia, são compostos por células adiposas maduras, sem qualquer evidência de atipia celular.^(1, 2)^ São mais comuns em mulheres de 30 a 60 anos e se apresentam clinicamente como uma massa móvel, circunscrita, de consistência macia e, em geral, indolor.^(2)^

Há dois tipos de lipomas, segundo Enzinger e Weiss:^(3)^ o superficial e o profundo. Os lipomas superficiais são geralmente encontrados em ombros, pescoço e região abdominal. Já os profundos são menos frequentes, localizam-se no mediastino anterior, na parede torácica e na região retroperitoneal, sendo classificados nos seguintes subtipos: intramuscular, intermuscular, parosteal e intraósseo.

Entre os lipomas profundos, os tipos intramuscular e intermuscular são os mais comuns; o lipoma parosteal é pouco frequente, e o intraósseo é raro.^(1)-4)^ O lipoma parosteal é um tumor profundo, de crescimento exofítico no periósteo, que pode causar compressão de nervos, erosão óssea ou hiperostose cortical focal. É um tumor raro, benigno e de crescimento lento, que representa 0,3% de todos os lipomas.^(1, 3, 4)^

Ao estudar 18.677 tumores mesenquimais, Kransdorf^(5)^ observou que 16% eram lipomas, sendo 1,3% intramuscular e 2,6% localizados no ombro e na axila, em região mais profunda.

Este relato de caso descreve uma paciente com diagnóstico de lipoma parosteal no úmero proximal, que desencadeou os sinais clínicos da síndrome do impacto. Nenhum caso semelhante foi encontrado em busca na literatura médica.

## RELATO DE CASO

Paciente do gênero feminino, 82 anos, com história de dor no ombro esquerdo há 2 anos e queixa de intensificação da dor nos últimos 2 meses. Procurou assistência médica com o início dos sintomas. O diagnóstico de tendinite do supraespinhoso foi feito com base no exame clínico e na ultrassonografia. Foi prescrita fisioterapia (analgesia e cinesioterapia) como tratamento conservador. Durante 18 meses, apresentou alternância de sintomas, com períodos de alívio ou intensificação.

Nos últimos 2 meses, a dor aumentou muito, com perda de capacidade funcional no membro superior esquerdo, que interferiu em suas atividades diárias. A elevação desse membro causava dor intensa, que a fazia despertar à noite muitas vezes.

Ao exame físico, a paciente apresentou hipotrofia leve do deltoide e, à inspeção dinâmica, a elevação ativa foi de 90° e a passiva foi de 160°. Observaram-se redução na força do músculo supraespinhoso, durante o teste de Jobe (*empty can test*),^(6)^ e sinal positivo no teste de Neer.^(7)^ Outros testes no exame físico e a amplitude de movimento na rotação lateral e medial foram comparados ao membro contralateral e considerados normais.

Após uma injeção de 10mL de xilocaína no espaço subacromial, a paciente sentiu alívio completo da dor e pode movimentar o ombro normalmente (teste de Neer positivo).^(7)^

Foram solicitadas radiografias em incidência posteroanterior do ombro esquerdo, axila e túnel do supraespinhoso. Foi relatado acrômio tipo II pela classificação de Bigliani.^(8)^

A ressonância magnética (RM) do ombro esquerdo (Figuras 1A e 1B) mostrou tendinite no supraespinhoso, sem ruptura do tendão do manguito do rotador, além de um grande tumor de tecido mole (provavelmente um lipoma), medindo 6,2x5,8x2,4cm, visível entre os ventres dos músculos deltoide, redondo menor e infraespinhoso.

**Figura 1:** (A) Corte sagital mostra lipoma subdeltoide, que não invade o espaço subacromial, e redução do tendão do supraespinhoso (seta). (B) Lipoma entre os músculos redondo menor (seta estreita) e deltoide (seta larga)

Foi indicada a excisão do tumor, que foi realizada por acesso posterior. O tumor de tecido mole não invadiu o espaço subacromial (Figura 2A). Sua extremidade superior estava no nível do acrômio posterior, e a inferior, aderida ao periósteo do úmero proximal, anterior à inserção do tendão dos músculos infraespinhoso e redondo menor (Figura 2B). Os vasos existentes no local e que penetravam a cápsula do tumor foram cauterizados.

**Figura 2:** (A) Imagem intraoperatória mostra que o lipoma (seta estreita) não invade o espaço subacromial (seta larga). (B) Lipoma aderindo ao úmero, anteriormente ao tendão dos músculos redondo menor (seta estreita) e infraespinhoso (seta larga)

Foi realizada exérese do tumor, com excisão das margens pela técnica *shelling out*, e o deltoide foi reinserido no acrômio posterior. A massa removida media 7,0x5,5x1,5cm (Figura 3A). O laudo do exame histológico foi lipoma parosteal (Figura 3B).

**Figura 3:** (A) Imagem do espécime cirúrgico. (B) Fotomicrografia do corte histológico do lipoma (hematoxilina e eosina, 200x)

No período pós-operatório, a paciente permaneceu com imobilização toracobraquial nas primeiras 6 semanas, e fazia apenas movimentos passivos de rotação lateral e medial. Depois, passou a fazer exercícios leves e ativos, sem resistência, e, após 2 meses, iniciou o fortalecimento muscular. A paciente esteve assintomática por 26 meses após a cirurgia.

## DISCUSSÃO

A função normal da estrutura muscular do ombro depende de alguns requisitos específicos, como integridade preservada e tônus dos músculos do manguito do rotador (supraespinhoso, infraespinhoso, redondo menor e subscapular), frouxidão capsular normal, arco coracoacromial íntegro, bursas delgadas e lubrificadas, e concentricidade das esferas de rotação glenoumeral e coracoacromial. As alterações desse intricado mecanismo são as causas mais comuns de problemas de dor nos ombros.^(9)^

O ombro é moldado por quatro barreiras anatômicas (espaço circunscrito por uma espessa parede de colágeno de fáscias e tendões, e os ossos): escápula com seus músculos; clavícula; úmero proximal; e o músculo deltoide. Nesses espaços, os tumores crescem na direção centrífuga, quando em contato com essas barreiras anatômicas.^(2)^ No caso relatado, o lipoma era do tipo parosteal e crescia no compartimento entre os músculos deltoide, infraespinhoso, redondo menor e o úmero.

O tumor desta paciente aumentava a pressão do compartimento nas contrações musculares, alterando a relação concêntrica das esferas de rotação. Em seu movimento de arco, a articulação entre o úmero e a escápula é formada por duas esferas concêntricas.

A esfera umeral é representada pela cabeça do úmero (com o menor raio), e a esfera do arco coracoacromial é formada pela superfície inferior do acrômio anterior e o ligamento coracoacromial (com o raio maior). As duas esferas têm o mesmo centro de rotação e juntas mantêm a melhor condição de estabilidade para o ombro e para a superfície disponível para transferir a carga escapular.^(9)^

A amplitude de movimento na elevação do braço, no plano escapular é de zero a 180°. Na fase inicial do movimento até 90°, a fixação da escápula ocorre sobre a parede torácica, pela contração dos músculos trapézio e serrátil anterior. Os músculos do manguito do rotador abaixam e fixam a cabeça do úmero na cavidade glenoide, e, ao mesmo tempo, o músculo deltoide, com predominância das porções média e posterior, atua ao levantar o braço. Quando o membro superior alcança uma elevação de 90°, a tuberosidade maior, para se movimentar por baixo do arco coracoacromial, requer a rotação externa do úmero, produzida pela contração dos músculos infraespinhoso e redondo menor. Esses músculos agem em sincronia, e produzem um movimento suave e coordenado.^(9)^ Neste caso, ao levantar seu braço, a sincronia na contração do deltoide, infraespinhoso e redondo menor é interrompida, devido ao lipoma interposto entre os músculos. O movimento perde sua coordenação e causa o impacto da tuberosidade maior contra o arco coracoacromial.

A ressecção do tumor restabeleceu a concentricidade das duas esferas de movimento (cabeça do úmero e acrômio anteriores, e arco coracoacromial) durante a rotação lateral nos graus finais de elevação. Isso ocorreu pela recuperação da congruência da articulação glenoumeral em seu arco completo de movimento, que levou ao alívio de sintomas da síndrome do impacto. A limitação deste relato de caso foi a falta de eletroneuromiografia, solicitada à paciente. Esse exame complementar poderia detectar a compressão do nervo axilar no espaço quadrangular, que mimetiza os sintomas de pacientes.

Para encontrar artigos que avaliassem pacientes com a síndrome subacromial relacionada ao lipoma, fizemos a busca em MEDLINE via PubMed (1966 a julho de 2013), EMBASE (1980 a 2013, semana 20) e LILACS (1982 a julho de 2013). Não houve limites de idiomas ou estado da publicação em nossa busca. Verificamos também as referências de artigos e revisões para outros estudos potencialmente relevantes. Nossa estratégia de busca combinou os termos específicos (“*shoul* *der impingement syndrome*” [Mesh] OU “*shoulder joint*” [Mesh] OU “*rotato* *r cuff*” [Mesh]) E “*Lipoma*” [Mesh] [“síndrome do impacto no ombro” OU “articulação do ombro” OU “manguito do rotador” E “lipoma”].

A estratégia de busca (finalizada em julho de 2013) identificou um total de 36 registros nos seguintes bancos de dados: PubMed (19), EMBASE (11), LILACS (6). O resultado da busca foi a identificação de seis artigos que avaliaram pacientes com doença de ombro associada à lipoma.^(10)-15)^

Rohrbough e Jobe^(10)^ relataram um caso de lipoma localizado na região do subdeltoide, que causava sintomas de instabilidade glenoumeral. Nisolle et al.^(11)^ apresentaram o caso de um homem de 44 anos com lipoma arborescente na bursa do ombro direito, associado à laceração do manguito do rotador. Relwani et al.^(12)^ descreveram o caso de uma paciente de 52 anos, com sinais clássicos de impacto no ombro; a paciente tinha um lipoma subacromial anterior que causava os sintomas. Os dois artigos de relato de caso ilustram dois casos de pacientes masculinos – 45 e 51 anos, com sinais e sintomas consistentes com síndrome do impacto, cuja etiologia era lipoma intramuscular que envolvia o músculo supraespinhoso.^(13, 14)^ Além disso, um relato de caso recente apresentou um paciente masculino, de 38 anos, com sintomas que mimetizavam tanto a instabilidade glenoumeral como o impacto subacromial,^(15)^ devido a um lipoma intermuscular subdeltoide. Em todos os casos, os sintomas desapareceram após a cirurgia e fisioterapia.

Consideramos nossa estratégia de busca abrangente, sem nenhuma restrição quanto a idiomas. Incluímos também busca manual (literatura cinzenta) e buscas por revisões e relatos de casos recentes e em andamento. Entretanto, é possível que tenhamos negligenciado alguns estudos potenciais que descreviam um caso semelhante. Portanto, em nosso entender, o relato deste artigo é um caso atípico de um lipoma parosteal do úmero proximal, que resultou em sinais e sintomas de impacto subacromial. O mecanismo que desencadeou os sintomas foi diagnosticado no pré-operatório por RM.
